# Salidroside Protects *Caenorhabditis elegans* Neurons from Polyglutamine-Mediated Toxicity by Reducing Oxidative Stress

**DOI:** 10.3390/molecules19067757

**Published:** 2014-06-10

**Authors:** Lingyun Xiao, Haifeng Li, Ju Zhang, Fan Yang, Aizhen Huang, Jingjing Deng, Ming Liang, Fangli Ma, Minghua Hu, Zebo Huang

**Affiliations:** 1 School of Pharmaceutical Sciences, Wuhan University, Wuhan 430071, China; E-Mails: xiaolingyun@whu.edu.cn (L.X.); lhf@whu.edu.cn (H.L.); juzi437163804@163.com (J.Z.); yangfan6616@126.com (F.Y.); cjhsusan@whu.edu.cn (J.D.); 2 Research & Development Centre, Infinitus (China) Company Ltd, Guangzhou 510665, China; E-Mails: fiona.liang@infinitus-int.com (M.L.); mary.ma@infinitus-int.com (F.M.); mandy.hu@infinitus-int.com (M.H.); 3 Guangdong Province Key Laboratory for Biotechnology Drug Candidates, School of Biosciences and Biopharmaceutics, Guangdong Pharmaceutical University, Guangzhou 510006, China; E-Mail: haz0709@163.com

**Keywords:** salidroside, polyglutamine, neurotoxicity, oxidative stress, *C. elegans*, chemoavoidance

## Abstract

Polyglutamine (polyQ) aggregation plays a pivotal role in the pathological process of Huntington’s disease and other polyQ disorders. Therefore, strategies aiming at restoring dysfunction and reducing stresses mediated by polyQ toxicity are of therapeutic interest for proteotoxicity diseases. Salidroside, a glycoside from *Rhodiola rosea*, has been shown to have a variety of bioactivities, including antioxidant activity. Using transgenic *Caenorhabditis elegans* models, we show here that salidroside is able to reduce neuronal death and behavioral dysfunction mediated by polyQ expressed in ASH neurons, but the neuroprotective effect is not associated with prevention of polyQ aggregation *per se*. Further experiments reveal that the neuroprotective effect of salidroside in *C. elegans* models involves its antioxidant capabilities, including decrease of ROS levels and paraquat-induced mortality, increase of antioxidant enzyme activities and reduction of lipid peroxidation. These results demonstrate that salidroside exerts its neuroprotective function against polyQ toxicity via oxidative stress pathways.

## 1. Introduction

Huntington’s disease (HD) is an age-related neurodegenerative disorder characterized by cognitive impairment and uncoordinated chorea, resulting from progressive loss of neuronal function [1]. The genetic mutation responsible for HD is the redundant CAG repeats (≥36), which encode elongated polyglutamine (polyQ) tracts within the mutant huntingtin protein [2]. It is well-known that polyQ and other pathogenic proteins (e.g., amyloid-β peptide; Aβ) associated with neurodegeneration often undergo conformational rearrangements and form insoluble aggregate deposits, which disrupt normal functions of neurons and cause damaging stresses such as transcription dysregulation, oxidative stress and ageing stress [3,4,5]. Therefore, strategies capable of inhibiting protein aggregation may have therapeutic potential for proteotoxicity disorders [6]. For example, epigallocatechin gallate (EGCG), the major polyphenol in green tea, is shown to reduce the cytotoxicity of huntingtin by modulating nucleation process in the early stage of protein aggregation [7]. Intriguingly, however, inhibition of protein aggregation alone may not be adequate to cure neurodegenerative disorders—immunization of Alzheimer’s disease patients with Aβ42 can clear amyloid plaques in the brains, but fails to prevent neurodegenerative symptoms [8]—suggesting detrimental effects caused by protein aggregation, rather than protein aggregates *per se*, may be more important. Taking oxidative stress as an example, high level of reactive oxygen species (ROS), which damages cellular components and triggers apoptosis, is observed in the neurons of HD patients and HD-like animal models [9,10]. Hence, reducing oxidative stress and enhancing stress resistance via genetic manipulation or chemical intervention are potentially capable of attenuating polyQ toxicity. For example, grape seed phenolic extract can effectively reduce ROS and ameliorate spatial memory impairment in HD mice [11], while the antioxidant coenzyme Q10 is able to improve the motor performance and reduce weight loss in HD mice [12].

The nematode *Caenorhabditis elegans* is a widely used model animal and is powerful at the molecular as well as organismal levels due to its small size, transparent body, ease of manipulation and rich genetic resources. Although *C. elegans* is a tiny organism (~1 mm in length), it contains about two-thirds of the potential counterparts of human disease genes and nearly one-third of its somatic cells are neurons [13]. Along with its short lifespan, genetic tractability and multiple behavioral phenotypes, this nematode model also offers special advantages in studies of age-onset neurodegenerative disorders [14]. For example, the pan-neuronal expression of human Aβ42 protein in *C. elegans* strain CL2355 causes behavioral deficits in chemotaxis, associative learning and thrashing [15]. In addition to age-related disease pathologies, *C. elegans* has also been used to evaluate potential anti-neurodegenerative therapeutics and their underlying mechanisms [14,16,17]. We have, for instance, demonstrated that astragalan, a polysaccharide from the medicinal plant *Astragalus membranaceus*, not only reduces polyQ aggregation and neurotoxicity but also extends lifespan of both wild-type and polyQ nematodes [18].

Salidroside is a phenol glycoside from the medicinal plant *Rhodiola rosea*, which grows in high mountains and has long been used as an anti-fatigue herb in China. Recent studies have revealed that salidroside has a variety of bioactivities in cultured cells, including neuroprotection, anti-oxidation, anti-ageing, anti-inflammation and anti-tumor effects [19,20,21,22]. For example, salidroside is shown to attenuate cell death induced by exogenous glutamate in cultured hippocampal neurons of rats [23], suggesting its neuroprotection potential. However, the neuroprotective effect of salidroside and the underlying mechanisms at organismal level is lacking. In this study, therefore, we investigated the protective effect of salidroside on behavioral dysfunction in *C. elegans* models endogenously expressing polyQ, the key pathogenic protein leading to HD, and attempted to unravel the underlying mechanisms, including reduction of oxidative stress.

## 2. Results and Discussion

### 2.1. Salidroside Prevents PolyQ-Mediated Neuronal Death in C. elegans

**Figure 1:**
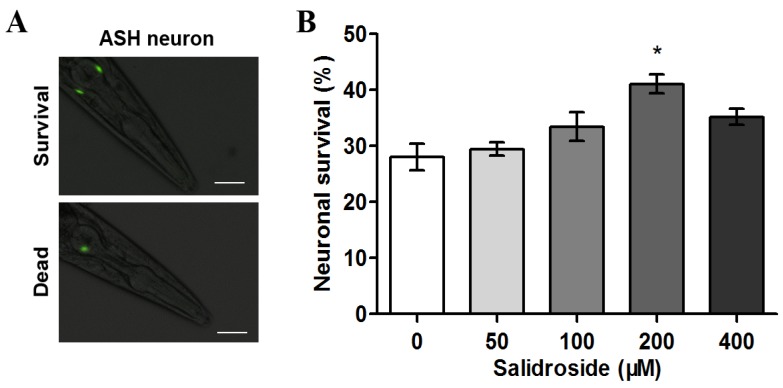
Effect of salidroside on the survival of ASH neurons in *C. elegans* HA759. (**A**) Micrographs of HA759 nematodes expressing Htt-Q150 in ASH neurons. Death of ASH neurons is assessed by loss of bilateral GFP fluorescence. Scale bars, 20 μm; (**B**) Survival rate of ASH neurons after salidroside treatment. The nematodes were treated with salidroside from L1 at indicated concentrations at 20 °C for 72 h and then subjected to neuronal survival assay. Data are representative of three independent experiments and presented as mean ± SD. *p < 0.05.

The transgenic *C. elegans* strain HA759 expresses Htt-Q150 (a polyQ150 tract derived from human huntingtin) strongly in ASH neurons and weakly in other neurons, leading to ASH neuronal death [24]. Using GFP fluorescence as an indicator for the survival of ASH neurons, we tested the protective effect of salidroside against polyQ-mediated neurotoxicity. As shown in Figure 1, only 28% of ASH neurons survived in untreated nematodes after three days, indicating significant neuronal death induced by the expression of polyQ tracts. When the nematodes were treated with 200 μM of salidroside, the neuronal survival rate increased to 41%, demonstrating that salidroside is capable fo reducing polyQ-mediated neuronal death.

### 2.2. Salidroside Reduces PolyQ-Mediated Behavioral Dysfunction in C. elegans

**Figure 2:**
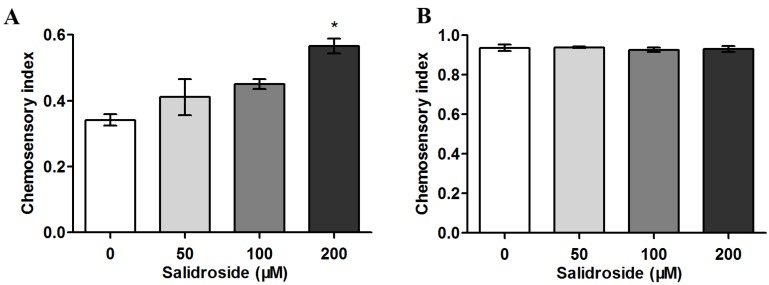
Effect of salidroside on chemosensory behavior in *C. elegans*. Nematodes were treated with salidroside from L1 at indicated concentrations at 20 °C for 72 h and then subjected to chemosensory assay. (**A**) HA759 nematodes. Data are presented as mean ± SEM of three independent experiments. * *p* < 0.05; (**B**) N2 nematodes. Data are representative of three independent experiments and presented as mean ± SD.

Since ASH neurons play an important role in the sensory response of *C. elegans* to aversive stimuli, loss of ASH function caused by polyQ expression makes the nematodes defective in chemoavoidance behavior [25]. Using HA759 nematode model, we have recently found that *Damnacanthus officinarum* extracts are able to alleviate polyQ-induced behavior deficiency [26]. To determine whether salidroside can rescue chemoavoidance dysfunction of *C. elegans*, HA759 nematodes were treated with salidroside at concentrations up to 200 μM based on the data from Figure 1 and then tested against high osmotic glycerol as described [14,26]. As shown in Figure 2A, the chemosensory index was ~0.35 in the untreated HA759 population, suggesting the majority of HA759 nematodes (~65%) lost the ability to sense osmotic glycerol due to ASH neuronal death. When treated with salidroside, the avoidance behavior of the nematodes was improved; at 200 μM, the chemosensory index was increased to ~0.6, indicating that ~60% of the nematodes have functional ASH neurons. As a control, wild-type N2 nematodes were also used in the assay and the chemosensory index was >0.9 in all the nematodes, indicating the healthy status of ASH neurons in >90% N2 nematodes either with or without salidroside treatment (Figure 2B). Together, these results demonstrate the protective effect of salidroside against neuronal dysfunction mediated by polyQ tracts.

### 2.3. Salidroside Does not Inhibit PolyQ Aggregation

**Figure 3:**
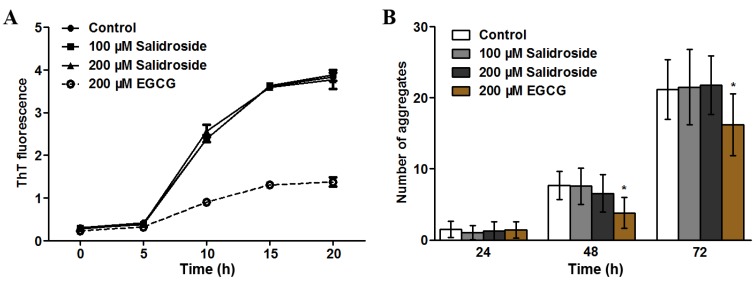
Effect of salidroside on polyQ aggregation. (**A**) Salidroside did not prevent *in vitro* polyQ aggregation. PolyQ40was first incubated with salidroside or EGCG at 37 °C for indicated times and then mixed with ThT prior to fluorescence determination; (**B**) Salidroside did not inhibit polyQ aggregation in *C.* *elegans*. AM141 nematodes were treated from L1 with salidroside or EGCG and scored for YFP positive aggregates at 24, 48 and 72 h, respectively. Data are representative of three independent experiments and presented as mean ± SD.

As polyQ plays a central role in HD pathogenesis and aggregation of polyQ proteins is closely associated with its toxicity, we examined whether salidroside exerts its protective effect through anti-aggregation. We first tested the effect of salidroside on polyQ aggregation *in vitro* using thioflavin-T (ThT) fluorescence assay [27]. As shown in Figure 3A, the ThT fluorescence in the control reaction was increased during the incubation time (0–20 h), which is consistent with previous studies (e.g., [28]). When salidroside was added, the increasing trend of ThT fluorescence was not altered, indicating that polyQ aggregation was not directly inhibited by salidroside (Figure 3A). In contrast, EGCG, a positive control against polyQ aggregation [29], significantly reduced ThT fluorescence (Figure 3A). Since organisms have a set of endogenous protein quality control system to maintain protein homeostasis in the presence of misfolded and aggregated proteins, we attempted to further investigate whether salidroside was capable of inhibiting polyQ aggregation *in vivo* using a transgenic polyQ nematode model. The *C. elegans* strain AM141 expresses polyQ40::YFP fusion proteins in body wall muscle cells and shows a discrete fluorescent aggregate phenotype when reaching adulthood [30]. We have previously demonstrated that astragalan is capable of reducing polyQ aggregation in this nematode model [18]. Therefore, we used this transgenic strain to test the effect of salidroside on polyQ aggregation *in vivo*. As shown in Figure 3B, however, salidroside did not affect the rapid formation of polyQ aggregates during the growth of AM141 nematodes, which was different from the action of the positive control EGCG. Interestingly, a previous study also demonstrates that salidroside can not inhibit the aggregation of Aβ42 proteins [31]. Taken together, our results suggest that anti-aggregation is not a major mechanism in the neuroprotective effect of salidroside.

### 2.4. Salidroside Decreases ROS Level and Increases Oxidative Survival in C. elegans

**Figure 4:**
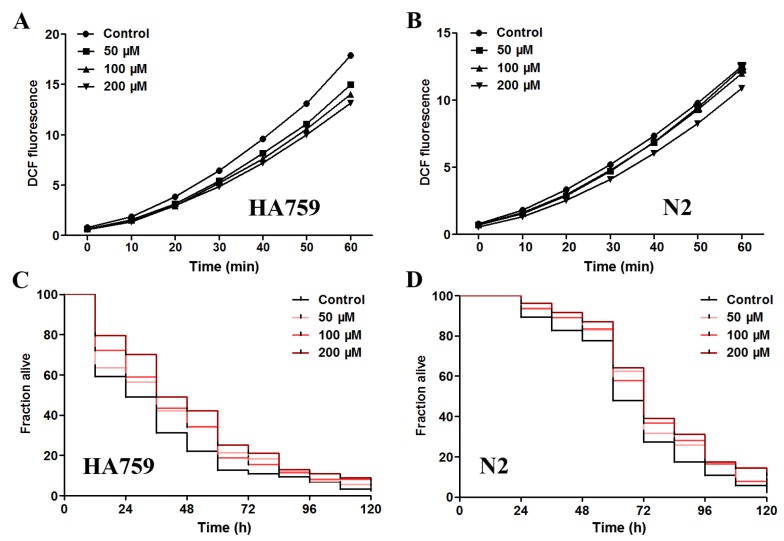
Effect of salidroside on ROS level and oxidative survival in *C. elegans*. (**A**, **B**) Salidroside decreased ROS level of HA759 and N2 nematodes. The nematodes were treated from L1 with salidroside at 20 °C for 72 h and then lysed for determination of ROS level by DCF assay every 10 min for 60 min. Data are representative of three independent experiments and presented as mean ± SD; (**C**, **D**) Salidroside increased the survival rates of paraquat-intoxicated polyQ HA759 and wild-type N2 nematodes, respectively. After treatment with salidroside at 20 °C for 48 h from young adult stage, the nematodes were exposed to 0.1 M paraquat and scored every 12 h for survival rate. Representative Kaplan–Meier survival curves are shown from three independent experiments.

Apart from protein aggregation itself, oxidative stress is another detrimental factor in many neurodegenerative diseases. For example, overexpression of mutant huntingtin can impair oxidative phosphorylation and increase the level of oxidative stress, leading to cell death [9]. Aβ has also been shown to increase ROS level and induce paralysis in *C.* *elegans* [32]. Therefore, reducing oxidative damage is a promising strategy against proteotoxicity. As salidroside is reported to modulate oxidative status in cell cultures inflicted by H_2_O_2_ [20], we examined its effect on ROS level in the transgenic polyQ nematode HA759 and the wild-type nematode N2 by DCF method. As shown in Figure 4A, the DCF fluorescence, which represents *in vivo* ROS levels, was decreased in HA759 nematodes after salidroside treatments (50–200 μM) as compared to the untreated nematodes, demonstrating the ROS clearance capacity of salidroside. In N2 nematodes, salidroside was also able to delay the increasing trend of DCF fluorescence at 200 μM (Figure 4B).

To further test whether salidroside can protect *C.* *elegans* against oxidative damage in general, we determined the survival curves of nematodes in the presence of paraquat, a potent ROS generator known to be highly toxic to animals and humans. As shown in Figure 4C,D, the survival rates of paraquat-intoxicated HA759 and wild-type N2 nematodes were clearly increased by salidroside at the tested concentrations (50–200 μM). These data indicate that salidroside is capable of reducing oxidative damages in *C. elegans*, which is in agreement with previous studies showing that salidroside was able to neutralize oxidative stress in cultured cells caused by other oxidant agents such as H_2_O_2_ [20]. Since neurodegeneration is closely associated with oxidative stress, these results suggest that the *in vivo* antioxidant activity of salidroside may play an important role in its protection against polyQ toxicity in HA759 nematodes. This is supported by a previous study showing that N-acetyl-l-cysteine can reduce polyQ-induced oxidative damages but does not decrease the polyQ inclusions in cell cultures [33].

### 2.5. Salidroside Increases Antioxidant Enzyme Activities and Inhibits Lipid Peroxidation in PolyQ Nematodes

It is known that oxidative stress caused by excessive ROS can be ameliorated by cellular antioxidant defense systems, which maintain ROS at optimal level either through prevention of oxidants from being formed or by removal of them. Therefore, we investigated the effect of salidroside on the activities of antioxidant enzymes superoxide dismutase (SOD) and catalase (CAT) and the content of lipid peroxidation product malondialdehyde (MDA). As shown in Table 1, the activities of SOD and CAT were significantly enhanced in HA759 when the nematodes were treated with ≥100 μM salidroside for 3 days, while MDA content was significantly decreased when the nematodes were treated with 200 μM salidroside, as compared with untreated animals. In N2 nematodes, the activities of antioxidant enzymes were not changed after salidroside treatment while the MDA content was reduced (Table 1). Interestingly, a recent study has shown that salidroside is able to scavenge various ROS species *in vitro*, suggesting ROS scavenging capacity of salidroside also contributes to its antioxidant effect in HA759 nematodes [34]. These data suggest that salidroside exerts its antioxidant protection in polyQ nematodes by increasing the activity of antioxidant enzymes and reducing lipid peroxidation, which is consistent with a previous study demonstrating the antioxidant capability of salidroside against oxidative insults in human fibroblast cell cultures [20]. A similar example is EGb761, which is found to have greater capacity on scavenging free radicals in AD-related *C. elegans* than in wild-type nematodes [35]. Taken together, our results suggest that salidroside is able to inhibit polyQ-mediated neurotoxicity through its antioxidant ability.

**Table 1:** Effect of salidroside on the activity of antioxidant enzymes and malondialdehyde content in *C. elegans*.

Strain	Salidroside (μM)	Antioxidant Enzyme Activity ^a^		MDA Content ^b^
		SOD	CAT	
HA759	0	16.2 ± 0.35	870 ± 78	0.66 ± 0.037
	50	20.6 ± 1.37	1104 ± 74	0.51 ± 0.084
	100	21.5 ± 1.78 ^c^	1427 ± 188 ^c^	0.45 ± 0.14
	200	21.3 ± 0.63 ^c^	1418 ± 37 ^c^	0.24 ± 0.031 ^c^
N2	0	19.01 ± 0.24	1147 ± 26	0.59 ± 0.060
	50	19.42 ± 0.78	1063 ± 80	0.45 ± 0.15
	100	18.30 ± 0.62	1066 ± 90	0.43 ±0.061
	200	20.06 ± 0.90	1176 ± 11	0.30 ± 0.059^ c^

^a^ SOD, U/mg protein; CAT, U/μg protein; ^b^ MDA, μM/mg; ^c^*p* < 0.05.

## 3. Experimental Section

### 3.1. Chemicals and Materials

Salidroside and paraquat were purchased from Aladdin (Shanghai, China). 5-Fluoro-2'-deoxyuridine (FUdR), 2',7'-dichlorofluorescin diacetate (DCFH-DA), and thioflavin-T (ThT) were purchased from Sigma (St. Louis, MO, USA). SOD, CAT, MDA and BCA assay kits were purchased from Beyotime (Haimen, China).

### 3.2. Strains and Maintenance

The nematode strains N2 (wild type), HA759 {*rtIs11*[*osm-10p*::*GFP*+*osm-10p::HtnQ150*+*Dpy-20*(+)]} and AM141 {*rmIs133*[*unc-54p*::*Q40*::*YFP*]} were obtained from the *Caenorhabditis* Genetics Center (University of Minnesota, St Paul, MN, USA). The nematodes were cultured at 20 °C on NGM agar plates seeded with *E. coli* OP50 as food. Synchronized eggs were prepared using sodium hypochlorite method, and allowed to hatch at 20 °C for 24 h with gentle shaking before any treatment.

### 3.3. Neuronal Survival Assay

*C. elegans* strain HA759 was used for neuronal survival assay as described [18]. Briefly, synchronized L1 larvae were treated with salidroside at 20 °C for 3 days and then the nematodes were collected, paralysed with 2% NaN_3_ and mounted on a 2% agarose pad. About 50 randomly selected nematodes were scored for GFP-positive ASH neurons using a BX51 fluorescence microscope (400×, Olympus, Tokyo, Japan).

### 3.4. Chemosensory Assay

Chemosensory assay was performed as previously described [14,26]. Briefly, about 300 synchronized L1 larvae were treated with salidroside at 20 °C for 3 days in S. medium with OP50 as food. The nematodes were washed three times with M9 buffer to remove bacteria and resuspended in M9 buffer. Before chemosensory assay, the 9 cm NGM plates were divided into two areas (A and B) with a 8 M glycerol line in the middle. Then 1% butanedione (2 μL) was added to the plate edge of area A to attract the nematodes, and 200 mM NaN_3_ (2 μL) was dropped near the butanedione to paralyze any attracted nematodes. About 300 synchronized adult nematodes in M9 buffer (20 μL) were dropped in the middle of area B. After incubation at 23 °C for 90 min, the nematodes in areas A and B were counted and calculated for chemosensory index as B/(A + B).

### 3.5. PolyQ Aggregation Assay

Preparation of GST-polyQ40 and *in vitro* polyQ aggregation assay were performed as described previously [28,36]. Briefly, 2 mg/mL of GST-polyQ40 was digested by 0.001% trypsin in PBS overnight at 37 °C to cleave the GST tag. The mixture was then boiled for 10 min to denature trypsin. After centrifugation, the supernatant was lyophilized and dissolved in 10 mM Tris-HCl (pH 7.4). Protein content was quantified using BCA method according to the instructions of the manufacturer. Then polyQ40 (20 μM) was incubated with salidroside at 37 °C. At the end of indicated time, 10 μL of the incubation sample was mixed with 500 mM of Gly-NaOH (pH 7.4) and 100 μM ThT in a final volume of 100 μL, and measured for ThT fluorescence in a fluorescence microplate reader (Fluoroskan Ascent FL, Thermo, MA, USA) with an excitation 450 nm and an emission 480 nm.

*In vivo* polyQ aggregation assay was performed using *C. elegans* AM141 as described [18]. In brief, L1 larvae were treated with salidroside at 20 °C for indicated times. The nematodes were then collected, paralyzed with 20 mM NaN_3_ and mounted on a 2% agar pad. Approximately 50 animals were randomly selected in each treatment and scored for the number of polyQ40::YFP aggregates in muscle cells via an IX51 inverted fluorescence microscope (100×, Olympus, Tokyo, Japan).

### 3.6. ROS Level Measurement

The ROS level in nematodes was measured as previously described [15]. Synchronized L1 larvae were treated with salidroside at 20 °C for 3 days. The nematodes were then harvested and washed three times in M9 buffer. Approximately 500 animals were homogenized in 400 μL PBS with 0.1% Tween 20 at 4 °C using a glass homogenizer. After centrifugation, the supernatant was collected and subjected to protein quantification as above. Then, 200 μg/mL lysate was transferred into a 96-well plate and incubated with 50 μM of DCFH-DA. The fluorescence was measured using the fluorescence microplate reader every 10 min for 1 h with an excitation 485 nm and an emission 535 nm.

### 3.7. Paraquat Assay

The paraquat assay was performed as previously described [37]. Briefly, synchronized L1 larvae were cultured at 20 °C for 42 h and further incubated with 75 μg/mL of FUdR for 24 h. Then the young adults were treated with salidroside for 48 h before being exposed to 0.1 M of paraquat. After incubation for indicated times, the number of live or dead nematodes was scored based on their movement every 12 h until all animals were dead.

### 3.8. Determination of Antioxidant Enzyme Activity and MDA Content

About 2,000 nematodes were treated with salidroside at 20 °C for 3 days and then lysed in 350 μL lysis buffer. After homogenization and centrifugation as above, the supernatant was collected and used for determination of SOD activity [38], CAT activity [39] and MDA content [40] as described. Protein content was determined by BCA method. The enzyme activities and MDA content were normalized by protein content.

### 3.9. Statistic Analysis

Statistical analysis was performed using one-way analysis of variance (ANOVA) in multiple group comparisons. *C. elegans* survival data were analyzed by Kaplan–Meier method and Peto’s log-rank test. Probability values of *p* < 0.05 were considered to be significant.

## 4. Conclusions

Using transgenic polyQ *C. elegans* models, we report in this study that salidroside is capable of reducing neuronal death and chemoavoidance dysfunction induced by polyQ expression in ASH neurons. However, the neuroprotective effect of salidroside is not associated with direct inhibition of polyQ aggregation. We further show that salidroside is not only able to reduce ROS levels and increase the survival rates of paraquat-intoxicated nematodes but also able to increase the activities of antioxidant enzymes and decrease lipid peroxidation level in the polyQ nematodes. Our study demonstrates that salidroside can protect neurons from polyQ-mediated toxicity by reducing oxidative stress in *C. elegans* models.
